# Cell-associated bacteria in the human lung microbiome

**DOI:** 10.1186/2049-2618-2-28

**Published:** 2014-08-18

**Authors:** Robert P Dickson, John R Erb-Downward, Hallie C Prescott, Fernando J Martinez, Jeffrey L Curtis, Vibha N Lama, Gary B Huffnagle

**Affiliations:** 1 Division of Pulmonary and Critical Care Medicine, Department of Internal Medicine, University of Michigan Medical School, Ann Arbor, MI 48109, USA; 2 Pulmonary and Critical Care Medicine Section, Medical Service, VA Ann Arbor Healthcare System, Ann Arbor, MI 48105, USA; 3 Department of Microbiology and Immunology, University of Michigan Medical School, Ann Arbor, MI 48109, USA

**Keywords:** Lung microbiome, Bronchoalveolar lavage, 16S, Pyrosequencing, Pneumonia

## Abstract

**Background:**

Recent studies have revealed that bronchoalveolar lavage (BAL) fluid contains previously unappreciated communities of bacteria. *In vitro* and *in vivo* studies have shown that host inflammatory signals prompt bacteria to disperse from cell-associated biofilms and adopt a virulent free-living phenotype. The proportion of the lung microbiota that is cell-associated is unknown.

**Results:**

Forty-six BAL specimens were obtained from lung transplant recipients and divided into two aliquots: ‘whole BAL’ and ‘acellular BAL,’ the latter processed with a low-speed, short-duration centrifugation step. Both aliquots were analyzed via bacterial 16S rRNA gene pyrosequencing. The BAL specimens represented a wide spectrum of lung health, ranging from healthy and asymptomatic to acutely infected. Bacterial signal was detected in 52% of acellular BAL aliquots, fewer than were detected in whole BAL (96%, *p ≤* 0.0001). Detection of bacteria in acellular BAL was associated with indices of acute infection [BAL neutrophilia, high total bacterial (16S) DNA, low community diversity, *p* < 0.01 for all] and, independently, with low relative abundance of specific taxonomic groups (*p <* 0.05). When whole and acellular aliquots from the same bronchoscopy were directly compared, acellular BAL contained fewer bacterial species (*p* < 0.05); whole and acellular BAL similarity was positively associated with evidence of infection and negatively associated with relative abundance of several prominent taxa (*p <* 0.001). Acellular BAL contained decreased relative abundance of *Prevotella* spp. (*p <* 0.05) and *Pseudomonas fluorescens* (*p <* 0.05).

**Conclusions:**

We present a novel methodological and analytical approach to the localization of lung microbiota and show that prominent members of the lung microbiome are cell-associated, potentially via biofilms, cell adhesion, or intracellularity.

## Background

Novel techniques of culture-independent microbial identification have revealed that bronchoalveolar lavage (BAL) fluid acquired from healthy and diseased subjects contains diverse communities of previously unappreciated bacteria [1-4]. The location of these bacteria within the various compartments of the respiratory tract is unknown. Recent *in vitro* and *in vivo* studies have demonstrated that some biofilm-associated bacteria, when stimulated by inflammation-associated host signals (fever, norepinephrine, free ATP), disperse from biofilms and adopt a planktonic phenotype with increased virulence [5].

Most lung microbiome studies to date have employed pyrosequencing of 16S rRNA gene amplicons obtained from whole BAL specimens [1], while others have used acellular BAL obtained via a low-speed, short-duration centrifugation step for eukaryotic cell removal [6,7]. This use of acellular BAL may exclude bacteria that are cell-associated via biofilms, cell-adhesion appendages, or intracellularity, though to date no published study has directly compared whole BAL to acellular BAL microbiota.

In this study, we sought to determine which members of the lung microbiome are predominantly cell-associated and which are free-living within the respiratory tract. We hypothesized that removal of eukaryotic cells from BAL fluid would alter the composition of the microbial communities identified by pyrosequencing, reflecting the selective removal of cell-associated bacteria. We further hypothesized that the predominance of free-living bacteria would be associated with indices of acute infection. To test these hypotheses, we designed an analysis of 46 clinically obtained BAL specimens, each analyzed for bacterial community membership using both whole and acellular BAL. All BAL specimens were obtained from lung transplant recipients, which represented a wide spectrum of lung health (ranging from healthy and asymptomatic to acutely infected). The respiratory pathogen profile in this group is similar both to that observed in healthcare-associated pneumonia as well as pneumonia in other immunocompromised states [8-10]. We present a novel methodological and analytical approach to the localization of lung microbiota and demonstrate that prominent members of the lung microbiome are cell-associated.

## Results

### Factors associated with detection of bacteria in acellular BAL

As described in the ‘Methods’ section, 46 BAL specimens were obtained from lung transplant recipients, with 46% collected for an acute clinical indication (dyspnea, cough, radiographic infiltrate, or decline in lung function) and the remaining 54% performed as surveillance bronchoscopies on asymptomatic patients. As we have previously reported [11], the microbiological profile of respiratory pathogens identified in BAL obtained from symptomatic patients in our study strongly resembled that of healthcare-associated pneumonia/hospital-acquired pneumonia as well as pneumonia in other immunocompromised states [8,9], dominated by *Staphylococcus aureus*, *Pseudomonas aeruginosa*, and other non-fermenting gram negative rods. Each BAL specimen was divided into two equal aliquots, and eukaryotic cells were removed from one aliquot (‘acellular BAL’) by short, low-speed centrifugation. Both acellular and whole BAL aliquots were then centrifuged at high speed for 30 min to pellet the bacteria in the sample for DNA preparation, V3–V5 16S rRNA gene amplicon library generation, and 454 pyrosequencing and subsequent bioinformatics, as outlined in the ‘Methods’ section.

**Figure 1:**
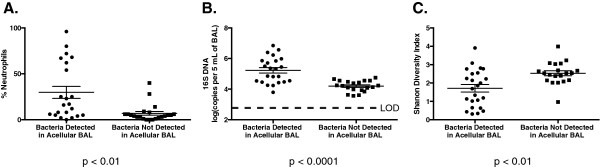
**Comparison of whole BAL specimens by whether bacterial DNA was detected in acellular specimens.** When whole BAL specimens were analyzed according to whether bacterial DNA was detected in their acellular fraction, specimens with bacterial DNA in the acellular fraction had more associated neutrophilia **(A)**, more bacterial (16S) DNA as measured by qPCR **(B)**, and lower community diversity as measured by the Shannon Index **(C)**. LOD, level of detection.

Bacterial communities were detected in 96% of whole BAL aliquots, while only 52% of acellular BAL aliquots contained detectable bacteria via pyrosequencing (*p ≤* 0.0001) (as assessed by the presence or absence of bands corresponding to the 16S rRNA gene after the amplification step of our pyrosequencing protocol). Given this marked difference, we compared BAL specimens based on whether their acellular aliquots contained detectable bacterial signal (Figure 1). Anticipating that total bacterial burden would be an important factor, we confirmed that samples with detectable bacteria in acellular BAL had higher bacterial (16S) DNA burden as measured by quantitative polymerase chain reaction (qPCR) of the whole BAL (*p* < 0.0001). Consistent with this, the same BAL specimens had higher BAL neutrophilia (*p* < 0.01) and lower whole BAL community diversity (*p* < 0.01), indicating that the presence of detectable bacteria in acellular BAL is associated with indices of acute infection.

**Figure 2:**
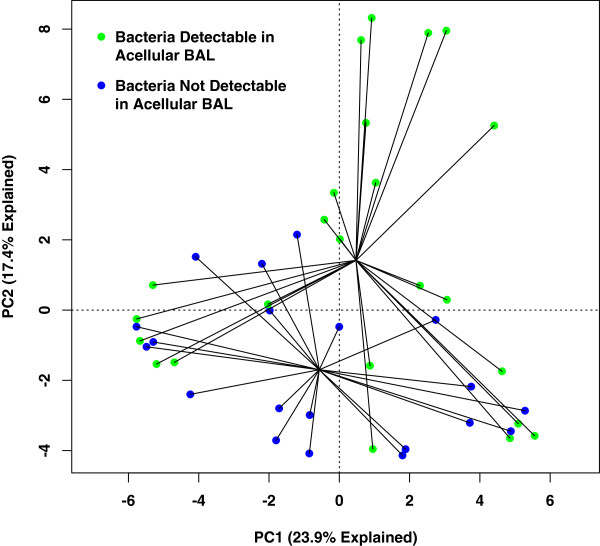
**Ordination analyses of bacterial communities detected in whole BAL samples.** Unsupervised principal component analysis (PCA) of bacterial communities in unfractionated whole BAL aliquots, as determined by pyrosequencing of V3–V5 16S rRNA gene amplicon libraries, labeled by whether bacteria could be detected in the acellular BAL aliquots of the same samples.

To compare the bacterial community membership of BAL specimens based on whether they had bacteria detectable in acellular BAL, we used the data visualization technique of unconstrained ordination, with whole BAL aliquots labeled according to whether bacteria were detectable in their corresponding acellular aliquots (Figure 2). This demonstrated a spatial separation of BAL specimens based on the presence of bacteria in their acellular aliquot, implying collective differences in the microbiota. This difference was confirmed as statistically significant using multiple methods of hypothesis testing [constrained ordination (redundancy analysis, RDA) and PERMANOVA (*adonis*), *p* < 0.05 both], even when controlled for total bacterial (16S) DNA (*p* < 0.05 both). These multiple analyses confirmed that the detection of bacteria in acellular specimens is independently influenced by bacterial community membership, not merely by total bacterial (16S) DNA.

**Figure 3:**
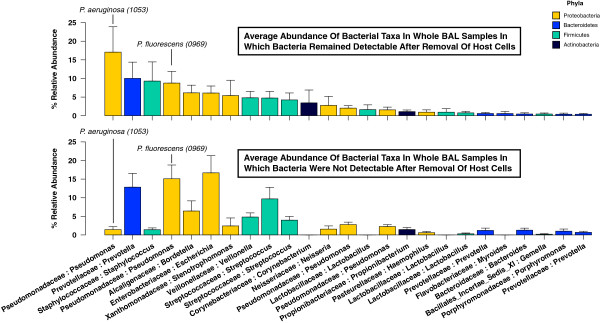
**Relative abundance of bacterial taxa detected in whole BAL samples.** The samples are divided according to whether bacteria were detected (top) or not (bottom) in the corresponding acellular BAL aliquot from the same specimen. The overall top 25 most abundant OTUs in the study are shown.

To better define the influence of community membership on detection of bacteria in the acellular BAL aliquot, we directly compared the relative abundance of the dominant taxa identified in whole BAL communities for each group. Figure 3 demonstrates the relative abundance of the most abundant operational taxonomic units (OTUs) detected in the specimens, ranked in descending order of mean abundance in specimens for which bacteria were detected in both whole and acellular BAL. Some prominent taxa associated with classic respiratory pathogens (*P. aeruginosa*, *Staphylococcus* sp.) were markedly less abundant in the specimens with undetectable bacteria in the acellular BAL aliquot. Other prominent taxa (*Prevotella* sp., *Pseudomonas fluorescens*, *Escherichia* sp.) had comparable or increased abundance in these same specimens. By direct comparison of group means, specimens with no bacteria detectable in their acellular BAL aliquot had significantly less *P. aeruginosa* and more *P. fluorescens* and *Escherichia* sp. (*p* < 0.05 for all). This further suggested that specific taxonomic groups are lost from BAL with the removal of eukaryotic cells.

In order to systematically identify and quantify the effects of factors on the detection of bacteria in acellular BAL, we performed univariate and multivariable logistic regression using host factors and whole BAL pyrosequencing results as predictors and detection of bacteria in acellular BAL as the outcome (Table 1). We again found that the detection of bacteria in acellular BAL by pyrosequencing was positively associated with indices of acute infection [total bacterial (16S) DNA, BAL neutrophilia and low community diversity (*p <* 0.01)]. Detection of bacteria in acellular BAL was negatively associated with relative abundance of the *Enterobacteriaceae* family (*p <* 0.05) and the OTU classified as *Escherichia* sp. (1087) (*p* < 0.05). Both of these taxonomic associations remained significant even when controlled via multivariable logistic regression for total bacterial (16S) DNA. Thus, detection of bacteria in acellular BAL aliquots was strongly associated with evidence of acute infection but also independently and negatively associated with relative abundance of specific prominent taxonomic groups, implying cell association of these types of microbes.

**Table 1:** Univariable logistic regression of predictors of bacterial identification

	**Predictors**	**Outcome: bacteria identified in acellular BAL via pyrosequencing**	
		***p *** **value**	**Odds ratio**
BAL features	Bacterial (16S) DNA (log)	*0.004*	*26.93 (4.58–476.29)*
	% Neutrophils	*0.027*	*1.06 (1.02–1.14)*
	% Lymphocytes	0.318	0.97 (0.91**–**1.02)
Antibiotics	Antibiotics (prior 30 days)	0.091	3.00 (0.86**–**11.29)
	Antibiotics (prior 7 days)	0.111	2.76 (0.81**–**10.14)
	Antibiotics (at time of BAL)	0.425	1.67 (0.48**–**6.10)
Diversity	Shannon Index	*0.009*	*0.31 (0.11–0.67)*
Phylum (% relative abundance)	Bacteroidetes	0.349	0.99 (0.95**–**1.02)
	Proteobacteria	0.933	1.00 (0.98**–**1.02)
	Firmicutes	0.601	1.01 (0.98**–**1.03)
Family (% relative abundance)	*Enterobacteriaceae*	*0.034*	*0.93 (0.86–0.99)*
	*Prevotellaceae*	0.494	0.99 (0.96**–**1.02)
	*Pseudomonadaceae*	0.367	1.01 (0.99**–**1.03)
	*Staphylococcaceae*	0.450	1.08 (0.99**–**1.39)
	*Streptococcaceae*	0.307	0.98 (0.93**–**1.02)
	*Veillonellaceae*	0.933	1.00 (0.91**–**1.09)
OTU (% relative abundance)	OTU 0969 (*P. fluorescens*)	0.194	0.97 (0.94**–**1.01)
	OTU 1053 (*P. aeruginosa*)	0.168	1.04 (1.00**–**1.19)
	OTU 1054 (*Bordetella*)	0.932	1.00 (0.94**–**1.06)
	OTU 1072 (*Streptococcus*)	0.172	0.96 (0.90**–**1.01)
	OTU 1077 (*Veillonella*)	0.999	1.00 (0.92**–**1.09)
	OTU 1087 (*Escherichia*)	*0.035*	*0.93 (0.87–0.99)*
	OTU 1095 (*Prevotella*)	0.625	0.99 (0.96**–**1.02)
	OTU 1098 (*Staphylococcus*)	0.442	1.07 (0.99**–**1.34)

Italicized results: *p* < 0.05.

### Influence of eukaryotic cell removal on BAL bacterial communities

**Figure 4:**
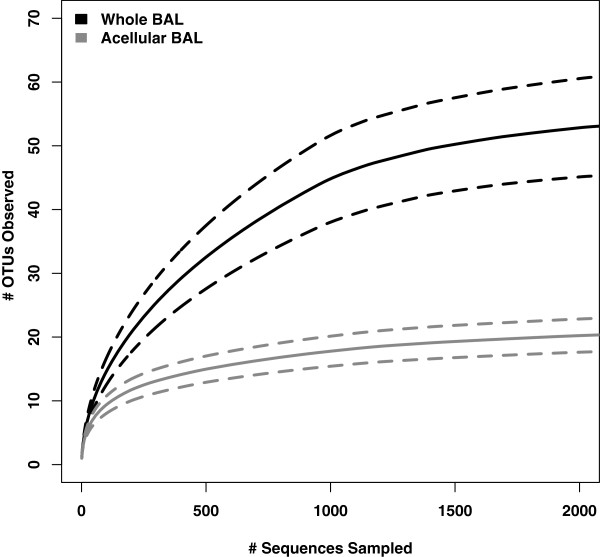
**Rarefaction curves for whole BAL and acellular BAL pyrosequencing results.** Rarefaction analysis was performed on whole BAL and acellular BAL aliquots obtained from the same BAL specimen. Dotted lines indicated 95% confidence intervals.

To determine the direct effect of host cell removal on detecting bacterial communities, we then focused on the 24 BAL specimens for which bacteria were detected in both whole and acellular aliquots. Twelve (50%) of these BAL specimens were acquired from symptomatic subjects; the remainder (12, 50%) were acquired from asymptomatic subjects. Similar numbers of high-quality pyrosequencing reads were obtained from both whole and acellular aliquots (1,633 ± 650 and 2,013 ± 1,287 reads per specimen, respectively, *p* > 0.05), but more unique OTUs were collectively detected among whole BAL aliquots (590 vs 267). Rarefaction analysis confirmed that lower OTU richness was decreased among acellular BAL aliquots when compared to whole BAL aliquots (Figure 4, *p* < 0.05). These results imply that specific bacterial species are lost from BAL specimens with removal of eukaryotic cells; the remaining OTUs may identify organisms that are dispersed or free-living within the respiratory tract.

**Figure 5:**
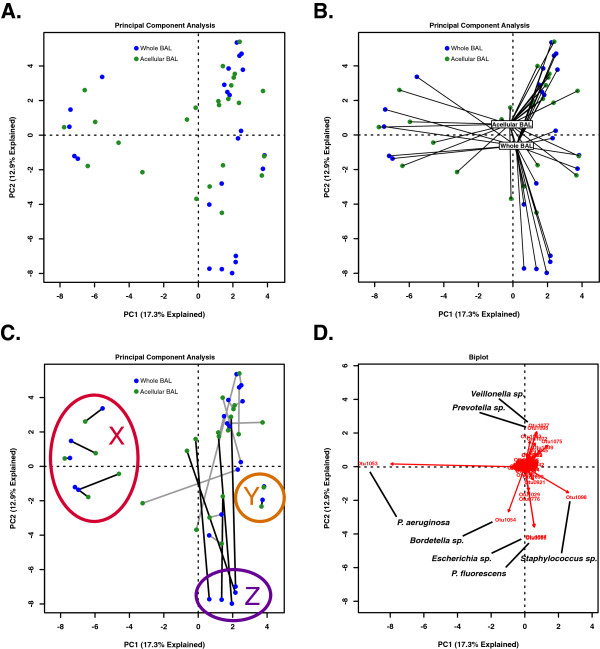
**Ordination of bacterial communities in whole and acellular BAL aliquots from the same BAL sample.** Unsupervised principal component analysis (PCA) labeled as **(A)** sample groups [whole BAL aliquots (blue) and acellular BAL aliquots (green)]; **(B)** sample groups with centroids added; **(C)** sample groups with corresponding whole and acellular BAL aliquots connected via lines. X and Y (red and yellow circles, respectively) are regions with close proximity of corresponding whole and acellular BAL aliquots; region Z (purple circle) contains whole BAL aliquots far removed from their acellular BAL counterparts. (See text.) **(D)** Biplot analysis of PCA plot with prominent OTUs labeled.

In order to compare the community membership of whole and acellular BAL aliquots from the same subjects, we again employed unconstrained ordination for data visualization (PCA, Figure 5A). We did not detect a clear and uniform spatial separation of whole and acellular BAL aliquots; centroids of each groups were close in proximity (Figure 5B), suggesting similar collective microbiota, and the two specimen groups were not statistically distinct when tested either via PERMANOVA (adonis) or constrained ordination (RDA) (*p <* 0.05 for both). Nevertheless, when examining the ordination distance separating the paired whole and acellular BAL communities derived from the same original BAL specimen, a marked heterogeneity between individual subjects was observed (Figure 5C). For some clusters of samples, whole BAL communities were similar to their acellular BAL counterparts (Figure 5C, regions X and Y). In other regions of ordination space, the whole and acellular BAL aliquots were consistently different from each other (Figure 5C, region Z). Using biplot analysis (Figure 5D), we determined that these regions were associated with specific OTUs. For instance, regions X and Y, in which the whole and acellular BAL communities of individual subjects were in close proximity to each other, were respectively associated with *P. aeruginosa* (1053) and *Staphylococcus* sp. (1098). By contrast, region Z, in which whole BAL communities were markedly dissimilar from their acellular BAL counterparts, was associated with *P. fluorescens* (0969) and *Escherichia* sp. (1087). Hence, the similarity of subjects' whole BAL pyrosequencing and acellular BAL pyrosequencing was related to community membership.

To quantify and better characterize this observation, we calculated a dissimilarity metric (the Bray-Curtis distance, calculated using a Hellinger-transformed data matrix) comparing each specimen's whole and acellular BAL bacterial communities. We then performed linear regression to test for associations between host and community factors and community dissimilarity (Table 2). The dissimilarity between whole and acellular BAL aliquots was negatively associated with total bacterial (16S) DNA (*p <* 0.0001), BAL neutrophilia (*p =* 0.01) and low community diversity (*p <* 0.0001), indicating that the aliquots tend to be similar in the context of acute infection. By contrast, dissimilarity between whole and acellular BAL aliquots was positively associated with relative abundance of the *Enterobacteriaceae* family (*p <* 0.001) and OTUs classified as a non-aeruginosa *Pseudomonas* sp. (0969) (*p <* 0.001) and *Escherichia* sp. (1087) (*p =* 0.001).

**Table 2:** Linear regression—BAL features and Bray-Curtis dissimilarity between paired whole and acellular BAL bacterial communities

	***R***^**2**^	**Slope**	***p *** **value**
Bacterial (16S) DNA	*0.5518*	*-0.3189*	*< 0.0001*
Shannon Diversity Index	*0.4563*	*0.2406*	*0.0003*
% Neutrophils	*0.2879*	*-0.006195*	*0.01*
Phylum: Bacteroidetes	0.154	0.006365	0.0579
Phylum: Proteobacteria	0.001186	-0.0003186	0.8731
Phylum: Firmicutes	0.0286	-0.001962	0.4295
Family: *Enterobacteriaceae*	*0.4015*	*0.3706*	*0.0009*
Family: *Prevotellaceae*	0.09077	0.004868	0.1525
Family: *Pseudomonadaceae*	0.0003322	0.0001943	0.9326
Family: *Staphylococcaceae*	0.1588	-0.005681	0.0538
Family: *Streptococcaceae*	0.03284	0.005290	0.3968
Family: *Veillonellaceae*	0.02873	0.007565	0.4285
OTU 0969: *P. fluorescens*	*0.4117*	*0.01502*	*0.0007*
OTU 1053: *P. aeruginosa*	0.1441	-0.004070	0.0673
OTU 1054: *Bordetella* sp.	0.007573	0.003108	0.686
OTU 1072: *Streptococcus* sp.	0.06459	0.01053	0.2308
OTU 1077: *Veillonella* sp.	0.02592	0.006867	0.4523
OTU 1087: *Escherichia* sp.	*0.3979*	*0.02464*	*0.001*
OTU 1095: *Prevotella* sp.	0.0658	0.004300	0.2263
OTU 1098: *Staphylococcus* sp.	0.1575	-0.005654	0.0549

Italicized results: *p* < 0.05.

**Figure 6:**
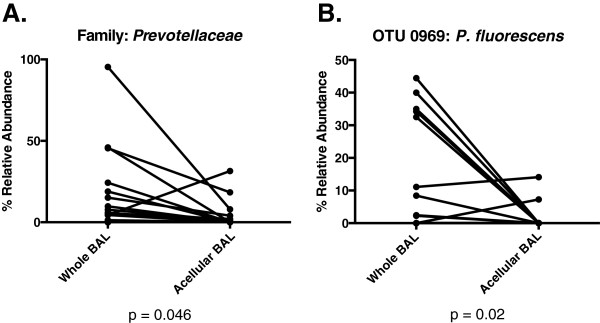
**Use of acellular BAL biases detection of microbial community composition via pyrosequencing.** Relative abundance of prominent lung microbes in paired whole and acellular BAL aliquots from the same BAL specimen: **(A)***Prevotellaceae* spp. and **(B)** OTU 0969 (*P. fluorescens*).

Given these results suggesting community membership-associated differences between whole and acellular BAL, we performed direct comparisons of the relative abundance of prominent taxonomic groups in paired whole and acellular BAL aliquots. We found marked differences in select (but not all) taxonomic groups. Acellular BAL samples contained significantly less *Prevotellaceae* family members (*p <* 0.05, Figure 6A) and *P. fluorescens* (*p* = 0.02, Figure 6B) than their whole BAL counterparts. Prominent taxa considered classic respiratory pathogens (*P. aeruginosa*, *Staphylococcus* sp., *Stenotrophomonas* sp.) had comparable relative abundance in paired whole and acellular specimens (*p* > 0.05). These data provide direct evidence that removal of eukaryotic cells alters the detection of microbial community composition in a taxonomically biased fashion, suggesting concomitant removal of cell-associated bacteria by this process.

## Discussion

Using a culture-independent technique of bacterial identification and multiple complementary analytic approaches, we found that the removal of host cells from BAL fluid both decreases its total bacterial content and also selectively alters its community composition, implying that specific bacterial community members are cell-associated. These novel findings have implications in the important biological question of localizing the lung microbiome, as well as in the study design of subsequent investigations and interpretation of existing literature of this relatively young field.

Via multiple analyses, we demonstrated that eukaryotic cell removal decreased the bacterial content of BAL fluid. The detection of bacterial communities via pyrosequencing was reduced by roughly half when acellular BAL was used. When whole and acellular BAL samples were directly compared, fewer unique OTUs were detected in acellular samples despite comparable numbers of high-quality reads, and rarefaction analysis confirmed a drop in taxonomic richness. Our analyses identified two important and distinct factors influencing loss of bacteria with host cell removal: the presence of acute infection [evidenced by high bacterial (16S) DNA burden, BAL neutrophilia and low community diversity] and, independently, relative abundance of specific prominent members of the lung microbiome. The association with infection may be attributable simply to an increased overall bacterial burden surviving the host-cell removal step of BAL processing but may also reflect the important transition from biofilm association to dispersed and free-living phenotype central to bacterial pathogenesis [5]. Separately, the association between abundance of specific taxa and loss of bacteria with host cell removal illustrates that the removal of eukaryotic cells did not uniformly decrease bacterial community membership, but instead removed bacteria in a taxonomically biased fashion.

This observation that eukaryotic cell removal selectively influences the composition of BAL bacterial communities was confirmed by direct comparison of whole and acellular BAL aliquots from the same bronchoscopy. Multiple complementary analyses—unconstrained and constrained ordination, linear regression using a community dissimilarity metric, and paired relative abundance comparison—all indicated that relative abundance of specific taxa (*Prevotella* spp., *P. fluorescens*, *Escherichia* sp.) is selectively decreased following eukaryotic cell removal. Several plausible biological explanations exist for this observation: these cell-associated bacteria may be intracellular (e.g., engulfed in alveolar macrophages), enmeshed in biofilms (all three of these taxa contain known biofilm producers) [12-14], or attached to eukaryotic cells via cell-adhesion appendages (all three taxa contain members with known means of attaching to or invading host epithelial cells [12,15,16]). Microbe localization techniques such as fluorescence *in situ* hybridization, electron microscopy, and biofilm-specific gene expression analysis may elucidate which of these conjectures are correct.

Our findings have important implications both in subsequent study design and in interpretation of the existing lung microbiome literature. While most studies to date have utilized whole BAL [1], some have utilized acellular BAL [6,7]. Our findings indicate that this methodological difference may have important effects on interpretation of BAL microbiota, especially in non-infected patients, and should be a consideration in the design of future studies. We demonstrated that in conditions of acute infection [BAL neutrophilia, high bacterial (16S) DNA burden, low community diversity], pyrosequencing of acellular BAL accurately and reliably reflects the composition of bacterial communities detected in whole BAL. Yet in other contexts, investigators utilizing acellular BAL should be mindful of the important consequences of host cell removal on bacterial community composition.

Our methodological approach has several caveats. The removal of eukaryotic cells via centrifugation is likely imperfect, and it is possible that the marked differences we found between whole and acellular BAL would be affected by variation in eukaryotic cell extraction technique. Second, our BAL specimens were all obtained from lung transplant recipients and thus are likely not completely representative of BAL acquired from healthy volunteers or of patients in other disease states. However, the microbiological profile of respiratory pathogens among transplant recipients does strongly resemble that in healthcare-associated pneumonia and pneumonia in other immunocompromised states [8,9], two of the most common indications for bronchoalveolar lavage [17].

## Conclusions

We present a novel methodological and analytical approach to the localization of lung microbiota and show that prominent members of the lung microbiome are cell-associated, potentially via biofilms, cell adhesion, or intracellularity.

## Methods

### Ethics statement

All clinical investigations were conducted according to the principles of the Declaration of Helsinki. The study protocol was approved by the institutional review board of the University of Michigan Healthcare System (HUM00042443). All patients provided written informed consent.

### Study population

BAL samples were obtained consecutively from lung transplant recipients undergoing bronchoscopy at the University of Michigan between 1 November 2011 and 1 August 2012. Clinical data were abstracted from the electronic medical record. We enrolled 33 subjects and performed 46 bronchoscopies, 21 (45.6%) for an acute clinical indication (dyspnea, cough, radiographic infiltrate, or decline in lung function) and the remainder as surveillance bronchoscopies performed on asymptomatic patients. Details of immunosuppression, antibiotic exposure, and comparison of symptomatic and asymptomatic subjects have been previously reported [11]. Compared to asymptomatic subjects, symptomatic subjects had comparable total bacterial (16S) DNA and community diversity but distinct community membership.

### Sample acquisition and processing

The bronchoscope was advanced via the mouth or nose and through the vocal cords. After a brief airway exam, the bronchoscope was wedged in the right middle lobe or lingula of the allograft (for surveillance bronchoscopies) or, in the case of symptomatic patients with available imaging, in the segment with the most evidence of radiographic abnormality. BAL was performed with instillation of between 120 and 300 ml of sterile isotonic saline. BAL cell and differential count was performed on pooled BAL fluid via hemocytometry by the University of Michigan Clinical Laboratory. All BALs were stored on ice until processing.

### Eukaryotic cell removal

BAL specimens were fractionated into two aliquots. One aliquot (acellular BAL) was centrifuged at 300 rpm (11 × *g*) for 10 min; the resulting supernatant was collected. This acellular supernatant and the other aliquot (whole BAL) were centrifuged at 13,000 revolutions per minute (rpm) (22,500 × *g*) for 30 min (Hermle Z 231 M microcentrifuge; Hermle Labortechnik GmbH, Wehingen, Germany) in dolphin-nosed Eppendorf tubes and the pellets for each stored at -80°C until the time of DNA extraction.

### DNA isolation, quantitative polymerase chain reaction, and 454 pyrosequencing

Genomic DNA was extracted from BAL pellets, resuspended in 360 μl ATL buffer (Qiagen DNeasy Blood & Tissue kit; Qiagen, Venlo, Limburg, the Netherlands) and homogenized in UltraClean fecal DNA bead tubes (MO-BIO, Carlsbad, CA, USA) using a modified protocol previously demonstrated to isolate bacterial DNA [18]. Quantification of bacterial 16S rDNA was performed on whole BAL specimens by real-time polymerase chain reaction (PCR) utilizing TaqMan hydrolysis probes on a Roche 480 LightCycler (Roche Diagnostics GmbH, Mannheim, Germany), as described previously [11,19-21]. Amplicon libraries were generated as previously described [11] and sequenced using a Roche 454 GS Junior according to established protocols [22]. The V3–V5 hypervariable regions of the bacterial 16S rRNA gene were sequenced in the V5–V3 direction using barcoded primer sets corresponding to 357 F and 926R [11]. Pre-procedure bronchoscopy rinse controls, reagent water controls, and mock community standards were sequenced and analyzed as quality controls. The data set supporting the results of this article has been posted to the NIH Sequence Read Archive (SRA:SRP041659).

### Data analysis

Sequence data were processed and analyzed using the software mothur v.1.27.0 according to the Standard Operating Procedure for 454 sequence data (http://www.mothur.org) using a minimum sequence length of 250 base pairs [23]. A shared community file and a phylotyped (genus-level grouping) file were generated using OTUs binned at 97% identity. OTUs detected in reagent water controls were removed from all BAL specimens prior to analysis. OTU numbers were arbitrarily assigned in the binning process and are referred to throughout the manuscript in association with their most specified level of taxonomy. Classification of OTUs was carried out using the mothur implementation of the Ribosomal Database Project (RDP) Classifier and the RDP taxonomy training set (http://rdp.cme.msu.edu). Using multiple complementary techniques (culture, microbe-specific PCR, NCBI BLAST, phylogenetic tree generation), we have previously identified two prominent *Pseudomonas*-classified OTUs in this dataset as *P. aeruginosa* (0153) and *P. fluorescens* (0969) [11]. Comparison of group proportions was performed using Fisher's exact test. Odds ratios were determined using univariable and multivariable logistic regression in *R*[24]. Group means were compared using Student's *t* test. Ordination and PERMANOVA (adonis) testing was performed using the vegan package 2.0-4 in *R*[24,25]. All analyses were performed in *R* and GraphPad Prism 6.

## Abbreviations

ATP: adenosine triphosphate; BAL: bronchoalveolar lavage; DNA: deoxyribonucleic acid; OTU: operational taxonomic units; PCA: principal components analysis; PCR: polymerase chain reaction; RDA: redundancy analysis; RDP: Ribosomal Database Project; rpm: revolutions per minute; rRNA: ribosomal ribonucleic acid.

## Competing interests

The authors declare that they have no competing interests.

## Authors’ contributions

RPD conceived of the study design, participated in data acquisition, executed the analysis and drafted and revised the manuscript. JRE participated in study design, data acquisition and bioinformatic analysis and revised the manuscript. HCP participated in statistical analysis and revised the manuscript. FJM participated in data acquisition, interpreted the data and revised the manuscript. JLC interpreted the data and revised the manuscript. VNL participated in study design and data acquisition, interpreted the data and revised the manuscript. GBH participated in study design, data acquisition, bioinformatic analysis and manuscript revision. All authors read and approved this version of the manuscript.
